# M2 macrophage-derived exosomes mitigate acute inflammation following ischemic stroke

**DOI:** 10.3389/fneur.2026.1733679

**Published:** 2026-02-04

**Authors:** Jinyang Song, Gang Su, Wei Chen, Xiaodong Xie, Zhenchang Zhang

**Affiliations:** 1 The Second Hospital & Clinical Medical School, Lanzhou University, Lanzhou, Gansu, China; 2 Institute of Genetics, School of Basic Medical Sciences, Lanzhou University, Lanzhou, Gansu, China

**Keywords:** exosomes, inflammation, ischemic stroke, microglia, Stat3, Syk

## Abstract

**Background:**

The acute inflammatory response following ischemic stroke is a key factor in exacerbating brain injury. Modulating excessive inflammation during the oxidative stress (OS) phase represents a potential therapeutic strategy; however, clinical interventions remain limited.

**Methods:**

M0 and M2 macrophage-derived exosomes (M0-exo and M2-exo) were administered to microglia under oxygen–glucose deprivation/reperfusion (OGD/R) conditions and to mice subjected to transient middle cerebral artery occlusion (tMCAO). The mechanisms underlying their anti-inflammatory effects were then investigated through a combination of bioinformatic analysis and fundamental experiments.

**Results:**

Treatment with exosomes markedly suppressed the expression of pro-inflammatory factors. Furthermore, they significantly reduced cerebral infarct volume and improved neurological function in mice. Notably, the anti-inflammatory effect of M2-exo was significantly superior to that of M0-exo. miRNA sequencing and subsequent validation revealed a specific enrichment of miR-330-5p in M2-exo. Mechanistic studies have demonstrated that miR-330-5p suppresses the expression of Spleen tyrosine kinase (Syk) and signal transducer and activator of transcription 3 (Stat3) in microglia, consequently reducing the production of downstream inflammatory factors. Treatment with Syk or Stat3 inhibitors partially mimicked the anti-inflammatory action of miR-330-5p in rescue studies.

**Conclusion:**

Our results unveil a novel anti-inflammatory pathway mediated by M2-exo, providing novel insights for stroke therapy.

## Graphical abstract

**Figure:**
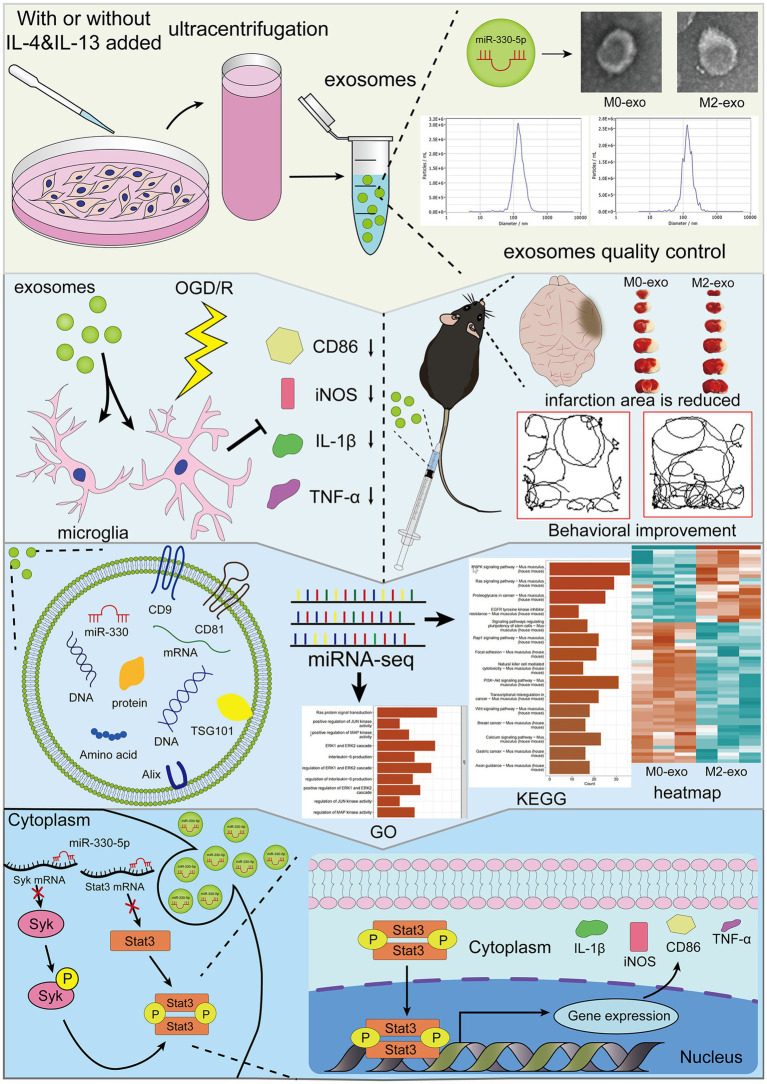

## 1 Introduction

Stroke represents a foremost cause of disability (1) and is responsible for the second highest number of deaths globally (2), posing a substantial burden on both patients and society. Ischemic stroke accounts for approximately 60–70% of all stroke cases (2, 3). Ischemia and hypoxia resulting from the interruption of local cerebral blood supply induce neuronal necrosis. The necrotic cells release damage-associated molecular patterns (DAMPs), which trigger the innate immune response and subsequently initiate the inflammatory process (4, 5). Although thrombolysis or thrombectomy within the therapeutic time window is currently an effective treatment (6), and reperfusion can salvage cells in the ischemic penumbra, it subsequently amplifies the inflammatory cascade after recanalization (7). While the inflammatory response plays a protective role, an excessive response can exacerbate brain injury. Attenuating this post-infarction hyperinflammation can mitigate brain damage (8). Given that microglia—the resident immune cells of the central nervous system—are the earliest activated and key drivers of the inflammatory response after stroke (9, 10), targeting their function to mitigate neuroinflammation has become a highly promising treatment strategy.

Spleen tyrosine kinase (Syk) is a critical molecule in innate immune signaling and plays a pivotal role in the progression of post-stroke inflammation (11). Studies have shown that the TREM1 receptor on microglia contributes to post-infarction inflammatory injury by activating Syk (12). Reducing the activation of Dectin-1/Syk can alleviate the post-infarction inflammatory response (13). The novel Syk inhibitor Bl1002494 attenuates arterial thrombosis and improves the prognosis of ischemic stroke (14), demonstrating significant therapeutic potential for Syk-based intervention. As another classical inflammatory signaling hub, the activation of signal transducer and activator of transcription 3 (Stat3) is equally crucial in the pathological process of cerebral infarction. Inhibition of the Jak2/Stat3 pathway can reduce the expression of the NLRP3 inflammasome in the post-ischemic inflammatory response (15). The Jak kinase inhibitor AG490 attenuates neurological deficits and diminishes infarct size in mice through suppression of the Jak2/Stat3 pathway (16). A growing number of studies demonstrate that numerous plant extracts impart neuroprotective effects after ischemic stroke by suppressing OS-induced inflammation through the inhibition of the Jak2/Stat3 pathway (17–21). It is particularly important that Syk activation is closely linked to Stat3 activation. Inhibiting PKM2 nuclear translocation can reduce the phosphorylation level of Stat3 in neutrophils, thereby alleviating the excessive inflammatory response and improving the outcome of ischemic stroke (22). Studies have demonstrated that during hepatic ischemia–reperfusion injury, Syk activation increases PKM2 nuclear translocation, which in turn enhances Stat3 phosphorylation, leading to the formation of neutrophil extracellular traps and ultimately exacerbating inflammatory damage after reperfusion (23). In microglia, PGLYRP1 exacerbates neuroinflammation via the TREM1–Syk–Erk1/2–Stat3 signaling axis (24). Therefore, modulating the activation of Syk and Stat3 in the early stages of cerebral ischemia represents a promising therapeutic strategy worthy of investigation for alleviating excessive inflammation.

Exosomes are cell-secreted, bilayer nanoscale vesicles (40–160 nm in diameter) which facilitate intercellular substance exchange and communication (25, 26). The inherent advantages of exosomes for the treatment of cerebral infarction stem from their low immunogenicity and high permeability across the blood–brain barrier (27). miRNAs constitute a critical component of the exosomal payload (28). Previous studies have demonstrated that the neuroprotective effect of M2 microglia-derived exosomes against ischemia–reperfusion injury is mediated through the anti-inflammatory action of miR-124 (29, 30). However, due to their rich cargo and diverse biological pathways, the mechanisms by which macrophage-derived exosomes exert their effects in ischemic stroke remain to be fully elucidated.

In our study, we explored the mechanism behind the anti-inflammatory action of M2-exo in response to oxidative stress (OS) injury, thereby revealing a new potential target for treating inflammation following ischemic infarction.

## 2 Materials and methods

### 2.1 Cell culture and M2 macrophage induction

The Raw264.7, BV2, and HEK293T cell lines were purchased from Procell (China). Cells were maintained in high-glucose DMEM (BasalMedia, China) containing 10% fetal bovine serum (FBS; ABW Nova Pharmaceutical Technology, China) and 1% penicillin/streptomycin (Gibco, USA). Cells were grown under standard humidified conditions (37 °C, 5% CO_2_). M2 macrophages were generated from Raw264.7 cells through a 24-h treatment with 20 ng/mL IL-4 and 20 ng/mL IL-13 (Gibco, USA).

### 2.2 Flow cytometry (FCM)

Raw264.7 cells were fixed and permeabilized according to the manufacturer’s instructions using the eBioscience™ Intracellular Fixation & Permeabilization Buffer Set (Invitrogen, USA). Fluorescently labeled primary antibodies, including anti-mouse CD206 antibody (0.8 μL per 100 μL, Invitrogen, USA) and IgG2b kappa Isotype Control (0.8 μL per 100 μL, Invitrogen, USA), were added at the recommended volumes. Analysis was performed using an Agilent NovoCyte Quanteon flow cytometer (USA).

### 2.3 Cell transfection

miR-330-5p mimics, miR-330-5p mimics negative control (MNC), miR-330-5p inhibitors, and miR-330-5p inhibitors negative control (INC) were obtained from GenePharma (China). Transfection of BV2 cells was performed using the siRNA-mate plus transfection reagent kit (GenePharma, China). BV2 cells were seeded in 6-well plates and transfected at approximately 60% confluence by adding a mixture of transfection buffer, miRNA (mimics, inhibitors, MNC, or INC), and siRNA-mate plus reagent to antibiotic-free culture medium, followed by a 24-h incubation under standard culture conditions.

### 2.4 Exosome isolation

Following a 24-h induction with IL-13 and IL-4, the complete medium for Raw264.7 cells was switched to serum-free basal medium (BasalMedia, China), and the cells were cultured for another 24 h. The supernatant was then collected, and exosomes were isolated via ultracentrifugation and resuspended in PBS. Exosome morphology was characterized by transmission electron microscopy (TEM; FEI, Czech Republic). Concentration and size distribution were analyzed using a ZetaView nanoparticle tracking analyzer (NTA; Particle Metrix, Germany), and exosome purity was assessed using a NanoCoulter analyzer (ResunTech, Shenzhen, China).

### 2.5 Exosome uptake

Cellular uptake of exosomes: PKH26, a red fluorescent dye with lipophilic properties, was used to label and trace exosomes by incorporating into their membranes (31). BV2 cells were seeded in 12-well plates. After cell adhesion, PKH26-labeled exosomes (20 μg/mL) were added to the culture and incubated for 24 h. Following fixation and permeabilization, cells were incubated with anti-Iba1 antibody (1:100, Cell Signaling Technology, USA), followed by an Alexa Fluor® 488-conjugated secondary antibody (Abcam, UK) to label the cytoplasm. Cell nuclei were counterstained with DAPI (Beyotime Biotechnology, China). Uptake of exosomes by BV2 cells was visualized using a fluorescence microscope (NIKON ECLIPSE Ti2-E, Japan).

Exosome uptake in mice: After 1.5 h of transient middle cerebral artery occlusion (tMCAO) induction and subsequent suture removal, DiR-labeled exosomes (7 μg/g body weight; AAT Bioquest, USA) were administered via tail vein injection. After 24 h, the distribution of exosomes in the mouse brain was examined using an *in vivo* imaging system (BIO-RAD, USA).

### 2.6 qRT-PCR for mRNA and miRNA

mRNA qRT-PCR: Total RNA was isolated from cultured cells and animal tissues with TRIzol reagent (Invitrogen, USA). Reverse transcription was performed to synthesize cDNA using the SweScript RT II First Strand cDNA Synthesis Kit (Servicebio, China). qRT-PCR was subsequently conducted using SYBR Green qPCR Master Mix (Servicebio, China) on a QuantStudio 3 Real-Time PCR System (Applied Biosystems, USA). The sequences of the mRNA primers are provided in Table 1.

**Table 1:** The sequences of the mRNA primers.

Gene	Forward primers (5′-3′)	Reverse primers (5′-3′)
CD206	CAAGGAAGGTTGGCATTTGT	CCTTTCAGTCCTTTGCAAGC
Arg1	CGCCTTTCTCAAAAGGACAG	CCAGCTCTTCATTGGCTTTC
CD86	AACGTATTGGAAGGAGATTACAGCT	CCTGCTAGGCTGATTCGGCT
iNOS	CAAGCACCTTGGAAGAGGAG	AAGGCCAAACACAGCATACC
TNF-α	ATGGCCTCCCTCTCAGTTC	TTGGTGGTTTGCTACGACGTG
IL-1β	CAACCAACAAGTGATATTCTCCATG	GATCCACACTCTCCAGCTGCA
SYK	GGCAACATCTCCAGAGATGAATC	GAGTAATGTTCCACTAGCTGCC
STAT3	CAATGGAGTACGTGCAGAAGACA	TGCAGCTCCTCCAGTTTCTTAAT
β-actin	GTGACGTTGACATCCGTAAAGA	GTAACAGTCCGCCTAGAAGCAC

miRNA qRT-PCR: cDNA synthesis for miRNA was carried out using the M5 Stem-loop miRNA cDNA Synthesis Kit (Mei5bio, China). The sequences of miRNA-specific primers are listed in Tables 2, 3. All subsequent steps for miRNA qRT-PCR followed the same procedure described above for mRNA.

**Table 2:** The sequences of miRNA-specific primers.

miRNA	Forward primers (5′-3′)	Reverse primers (5′-3′)
miR-15b-3p	AGCGCGAATCATTATTTGCT	GTTGTGGTTGGTTGGTTTGT
miR-199a-3p	GGGACAGTAGTCTGCACAT	GAGAGGAGAGGAAGAGGGAA
miR-223-3p	TGCGTGTCAGTTTGTCAAAT	GAGAGGAGAGGAAGAGGGAA
miR-212-3p	AGGGTAACAGTCTCCAGTCA	TCCTCCTCTCCTTCCTTCTC
miR-330-5p	GTCTCTGGGCCTGTGTC	GTTGTGGTTGGTTGGTTTGT
U6	CGCTTCGGCAGCACATATAC	CACGAATTTGCGTGTCATCC

**Table 3:** The sequences of RNA template linker sequence (RTL).

miRNA	RNA template linker sequence (RTL)
miR-15b-3p	GGTTGTGGTTGGTTGGTTTGTATACCACAACCTAGAGC
miR-199a-3p	GGAGAGGAGAGGAAGAGGGAAATCTCCTCTCCTAACCA
miR-223-3p	GGAGAGGAGAGGAAGAGGGAAATCTCCTCTCCTGGGGT
miR-212-3p	GTCCTCCTCTCCTTCCTTCTCATGAGGAGGACTGGCCG
miR-330-5p	GGTTGTGGTTGGTTGGTTTGTATACCACAACCGCCTAA

### 2.7 Western blot (WB)

Protein samples were resolved through electrophoresis with the aid of an SDS-PAGE Gel Kit (CWBIO, China). Both the electrophoresis and protein transfer steps were performed using a Mini-PROTEAN system (BIO-RAD, USA). After blocking with 5% skim milk (Solarbio, China), the membrane was incubated with primary antibodies overnight at 4 °C. The primary antibodies used were as follows: anti-CD206 (1:1000; Proteintech, China), anti-Arg1 (1:1000; Abmart, China), anti-Alix (1:6000; Proteintech, China), anti-TSG101 (1:800; Servicebio, China), anti-CD9 (1:1000; Affinity Biosciences, China), anti-Calnexin (1:1000; Affinity Biosciences, China), anti-CD81 (1:2000; Proteintech, China), anti-*β*-actin (1:5000; Proteintech, China), anti-iNOS (1:1000; Cell Signaling Technology, USA), anti-CD86 (1:500; Santa Cruz Biotechnology, USA), anti-IL-1β (1:2000; Proteintech, China), anti-TNF-*α* (1:1000; Proteintech, China), anti-Syk (1:1000; Proteintech, China), anti-p-Syk (1:1000; Bioss, China), anti-Stat3 (1:1000; BOSTER, China), and anti-p-Stat3 (1:1000; BOSTER, China). Protein bands were detected using an ECL chemiluminescent substrate kit (HUCH/WILBER, China) and visualized with a FUSION SOLO6S imaging system (VILBER, France).

### 2.8 OGD/R administration

When BV2 microglial cells reached 80% confluence, the culture medium was replaced with glucose-free medium (BasalMedia, China), and the cells were placed in a hypoxic environment (1% O_2_, 5% CO_2_, 94% N_2_) for 3 h in a triple-gas incubator (Thermo Fisher Scientific, USA) to induce oxygen–glucose deprivation (OGD). This was followed by a 12-h recovery period in complete medium under normoxic conditions.

### 2.9 Immunofluorescence (IF)

Cell immunofluorescence: Fixed BV2 cells were permeabilized with 0.2% Triton X-100 and blocked with 5% BSA. Cells were incubated overnight at 4 °C with a primary antibody against Arg1 (1:200; Abmart, China). To achieve fluorescence detection, the cells were exposed to an Alexa Fluor® 488-conjugated secondary antibody (Abcam, UK) for one hour at room temperature. Nuclei were counterstained with DAPI (Beyotime Biotechnology, China).

Brain section immunofluorescence: After dissection, brains were fixed in 4% paraformaldehyde for 24–48 h. The tissues were then cryoprotected in a graded sucrose series (20 and 30%). After dehydration, brains were embedded in OCT compound (SAKURA, USA), frozen at −20 °C, and coronally sectioned at 7 μm thickness using a cryostat (CM1950, Leica, Germany). Sections were incubated overnight at 4 °C with a mixture of primary antibodies against CD86 (1:100; Santa Cruz Biotechnology, USA) and Iba1 (1:100; Cell Signaling Technology, USA), applied at 50 μL per section. On the following day, a mixture of secondary antibodies—Alexa Fluor® 594-conjugated anti-mouse (1:500; Abcam, UK) and Alexa Fluor® 488-conjugated anti-rabbit (1:500; Abcam, UK)—was applied and incubated for 1 h at room temperature. All subsequent steps followed the same protocol described for cell immunofluorescence.

### 2.10 Histopathology

Immunohistochemistry: Mouse brains were immediately immersed in 4% paraformaldehyde for fixation over 48 h following dissection. A standard dehydration, clearing, and paraffin embedding workflow was carried out for the tissues using a Histocore Pearl tissue processor (Leica, Germany) and a YB-7LF embedding station (YAGUANG, China). Sections were cut at a thickness of 5 μm using a Leica RM2245 microtome (Leica, Germany). Slices were incubated overnight at 4 °C with a primary antibody against iNOS (1:600; Cell Signaling Technology, USA). On the following day, immunohistochemical detection was conducted using a universal two-step detection kit (PV-9000; Zhongshan Goldenbridge, China). Nissl staining and hematoxylin–eosin (H&E) staining were performed using a fully automated multifunctional staining system (Leica, Germany). All sections were scanned using a digital slide scanner (3DHISTECH, Hungary), and quantitative analysis was performed using Fiji software.^1^

### 2.11 Dual-luciferase reporter assay

DNA fragments (~200 bp) containing the predicted binding sites of Syk 3′- Untranslated Region (UTR)-Wild Type (WT), Syk 3′-UTR- Mutant type (Mut), Stat3 3′-UTR-WT, and Stat3 3′-UTR-Mut were synthesized and cloned into the pmirGLO dual-luciferase reporter vector. HEK293T cells were transfected using Lipofectamine 2000 (Gibco, USA). The recombinant pmirGLO plasmids were co-transfected with either miR-330-5p mimics or MNC into HEK293T cells. After 48 h, luciferase activity was measured using a dual-luciferase reporter assay kit (GENE CREAT, China).

### 2.12 Animals

Adult male C57BL/6 mice (aged 8–10 weeks, 25–30 g) were obtained from the Experimental Animal Center of Lanzhou University (Lanzhou, China; Laboratory Animal Certificate: SCXK (Gan) 2018–0002). Housed under standard conditions (12-h light/dark cycle, 20–25 °C, 40–60% humidity), the mice had free access to food and water. The Ethics Committee of the Second Hospital & Clinical Medical School, Lanzhou University, reviewed and approved all animal-related procedures (Approval Number: D2022-350).

### 2.13 tMCAO surgery and laser speckle imaging

Anesthesia in mice was induced by an intraperitoneal injection of tribromoethanol (JITIAN BIO, China). A midline cervical incision was made to expose the right external carotid artery (ECA) and common carotid artery (CCA) via blunt dissection. A monofilament suture (RWD, China) was inserted into the internal carotid artery via the ECA and advanced gently until resistance indicated occlusion of the middle cerebral artery. After 1.5 h of occlusion, the filament was withdrawn from the ECA to initiate reperfusion. The sham-operated group underwent identical surgical procedures with the omission of the suture insertion. A laser speckle contrast imaging system (RFLSI ZW; RWD, China) was used to measure cerebral blood flow before, during, and after reperfusion.

### 2.14 2,3,5-Triphenyltetrazolium chloride staining

Brains were coronally sectioned into 1.5-mm-thick slices. Sections were immersed in 2% (w/v) TTC solution (MedChemExpress, USA) in PBS and incubated in a 37 °C water bath for 20 min. Infarct volume percentages were quantified using Fiji software (see Footnote 1).

### 2.15 Stereotaxic injection

Stereotaxic injections were performed using the RWD 71000 fully automated stereotaxic system (RWD, China). Each mouse received 20 μg of miR-330-5p agomir or antagomir (GenePharma, China) delivered into the right striatum. Evans blue (1%; Sigma-Aldrich, USA) was administered to verify accurate targeting and assess injectate distribution after 24 h (19).

### 2.16 miRNA high-throughput sequencing and public database analysis

Exosomal miRNAs were profiled using high-throughput sequencing. The datasets GSE280846 and GSE248793 were sourced from the Gene Expression Omnibus database. Differential expression analysis, volcano plots, heatmaps, Gene Set Enrichment Analysis (GSEA), Gene Ontology (GO)/Kyoto Encyclopedia of Genes and Genomes (KEGG) enrichment analyses, and Venn diagrams were performed and visualized using R version 4.3.3.^2^ Detailed analysis scripts and plotting code are provided in Supplementary materials 1 (DOI: 10.6084/m9.figshare.30434296; https://figshare.com/s/b0b162b705ab91d1fdf6). Protein–protein interaction (PPI) and competing endogenous RNA (ceRNA) networks were constructed and analyzed using the STRING database^3^ and Cytoscape software,^4^ respectively.

### 2.17 Neurobehavioral tests

The Longa score was employed to evaluate neurological deficits (32). 0: no neurological deficit (normal); 1: incomplete extension of the contralateral forelimb (upon tail suspension); 2: circling to the contralateral side (during crawling); 3: falling to the contralateral side (during crawling); and 4: no spontaneous movement and loss of consciousness.

The open field test was conducted using the SmartV3.0 behavioral tracking system (Panlab, Spain) to record movement trajectory, distance traveled in the center zone, total resting time, and total distance traveled during a 3-min session in a square open field arena (100 cm × 100 cm).

Grip strength test: Each mouse was placed on the YLS-13A rodent grip strength meter (jnyiyan, China) and allowed to grip the horizontal bar with all four limbs. Mice were then gently pulled backward by the tail until they released the bar. The maximum grip force was recorded for statistical analysis.

Cylinder test: Forelimb use asymmetry was assessed by placing mice in a 5,000-mL glass beaker (SICHUAN SHUBO, China; 17 cm diameter, 27 cm height). During vertical exploration, the number of wall contacts made with each forelimb was recorded separately over 5 min. Only the initial weight-bearing contact of each limb was counted (33). The forelimb impairment score (%) was calculated as follows:

$[\begin{array}{l} (\begin{array}{l} \text{number of contacts contralateral to the infarct}+\\ 1/2\;\text{number of bilateral contacts} \end{array})/\\ \text{total contacts} \end{array}]\times 100\%$

Lower scores indicated more severe impairment.

Corner turn test: This test served to assess unilateral sensorimotor deficits in the stroke model (34). Healthy mice turned left and right with equal frequency, whereas tMCAO mice showed a preference for turning toward the infarcted side.

### 2.18 Statistical analysis

All statistical analyses were carried out with GraphPad Prism 10 (GraphPad Software, Inc., USA). Data are expressed as the mean ± standard deviation (SD). Normality was assessed using the Shapiro–Wilk test, and homogeneity of variance was evaluated with the Brown–Forsythe test. Neurological function scores were analyzed using the Kruskal–Wallis test. Comparisons between two groups were conducted using unpaired Student’s *t* tests, while one-way ANOVA was used for comparisons among multiple groups (*n* ≥ 3) involving a single independent variable. A *p*-value less than 0.05 was considered statistically significant.

## 3 Results

### 3.1 IL-4 and IL-13 in combination induce the polarization of M0 macrophages toward the M2 phenotype

**Figure 1:**
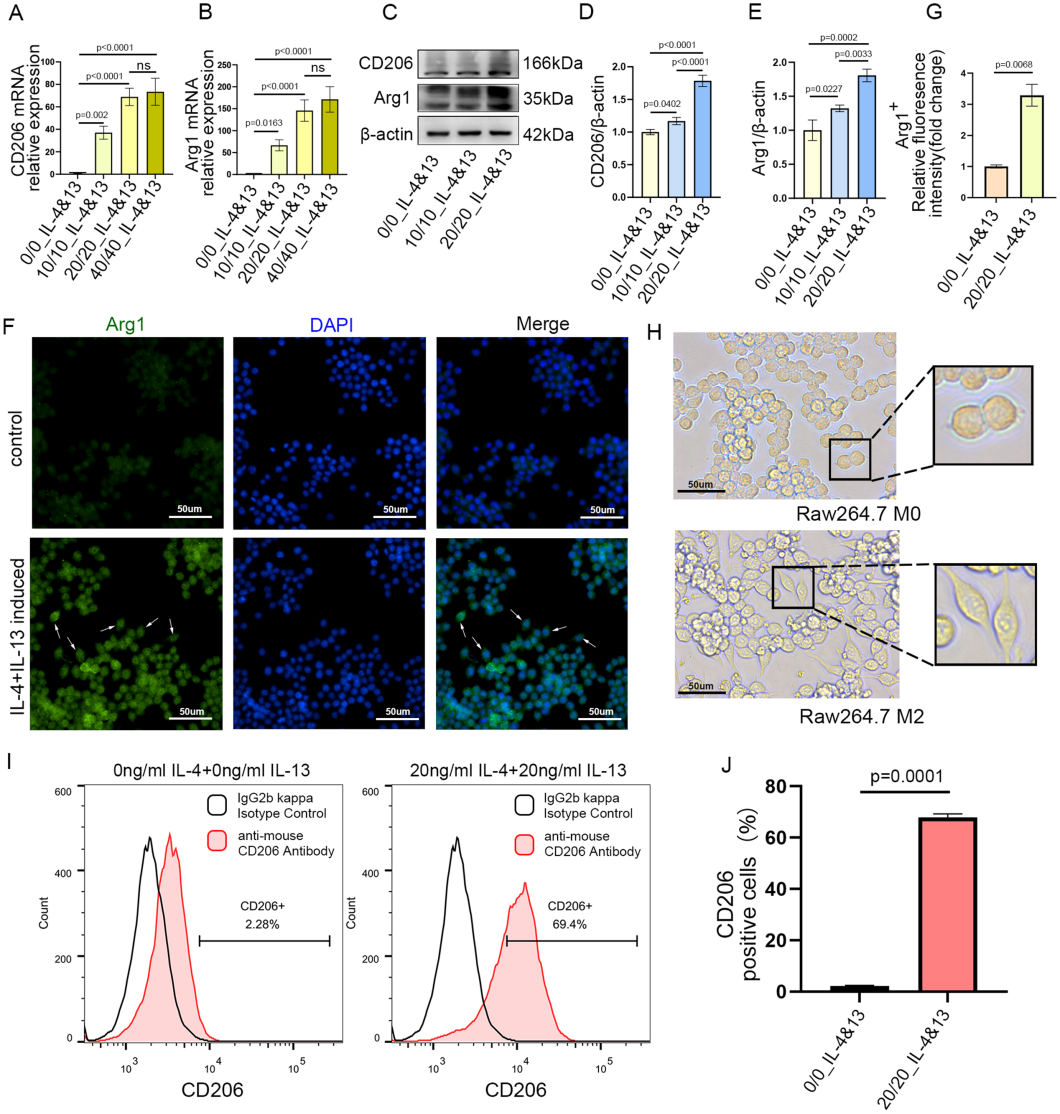
Raw264.7 cells were polarized into the M2 subtype by co-stimulation with IL-4 and IL-13 for 24 h. **(A,B)** Relative mRNA expression of CD206 and Arg1 in Raw264.7 cells after stimulation with IL-4 and IL-13 for 24 h (ng/mL). **(C–E)** Western blot analysis and quantification of CD206 and Arg1 expression (*n* = 3). **(F,G)** Arg1 immunofluorescence and corresponding quantitative analysis in control and treated cells. Scale bar = 50 μm (*n* = 3). **(H)** Morphological changes in Raw264.7 cells following treatment. Scale bar = 50 μm. **(I,J)** Flow cytometry analysis of the percentage of CD206-positive macrophages following induction with IL-4 and IL-13. Data are presented as mean ± SD.

To determine the optimal cytokine concentration for M2 polarization, a range of concentrations was tested. qRT-PCR analysis demonstrated that treatment with 20 ng/mL IL-4 plus 20 ng/mL IL-13 for 24 h significantly upregulated the expression of CD206 and Arg1 in Raw264.7 cells. Increasing the concentrations of IL-4 and IL-13 to 40 ng/mL did not result in a further substantial increase in CD206 or Arg1 expression. Based on these findings, 20 ng/mL IL-4 and 20 ng/mL IL-13 were selected as the standard conditioning protocol for M2 polarization (Figures 1A,B). The upregulation of CD206 and Arg1 protein expression was further confirmed by WB following stimulation with the same cytokine combination (Figures 1C–E). Immunofluorescence analysis revealed a markedly stronger Arg1 fluorescence signal in cells treated with IL-4 and IL-13 for 24 h compared to untreated controls (Figures 1F,G; Supplementary Figure 1). Morphological differences between M0 and M2 macrophages were observed under light microscopy. M0 macrophages exhibited a spherical or oval shape, whereas M2 macrophages displayed an elongated, spindle-like morphology (Figure 1H). Finally, FCM analysis confirmed that IL-4 and IL-13 co-stimulation significantly elevated the proportion of CD206-positive cells in Raw264.7 macrophages (Figures 1I,J). Taken together, these results demonstrate the successful polarization of M0 macrophages toward an M2 phenotype.

### 3.2 Exosome characterization and uptake *in vivo* and *in vitro*

**Figure 2:**
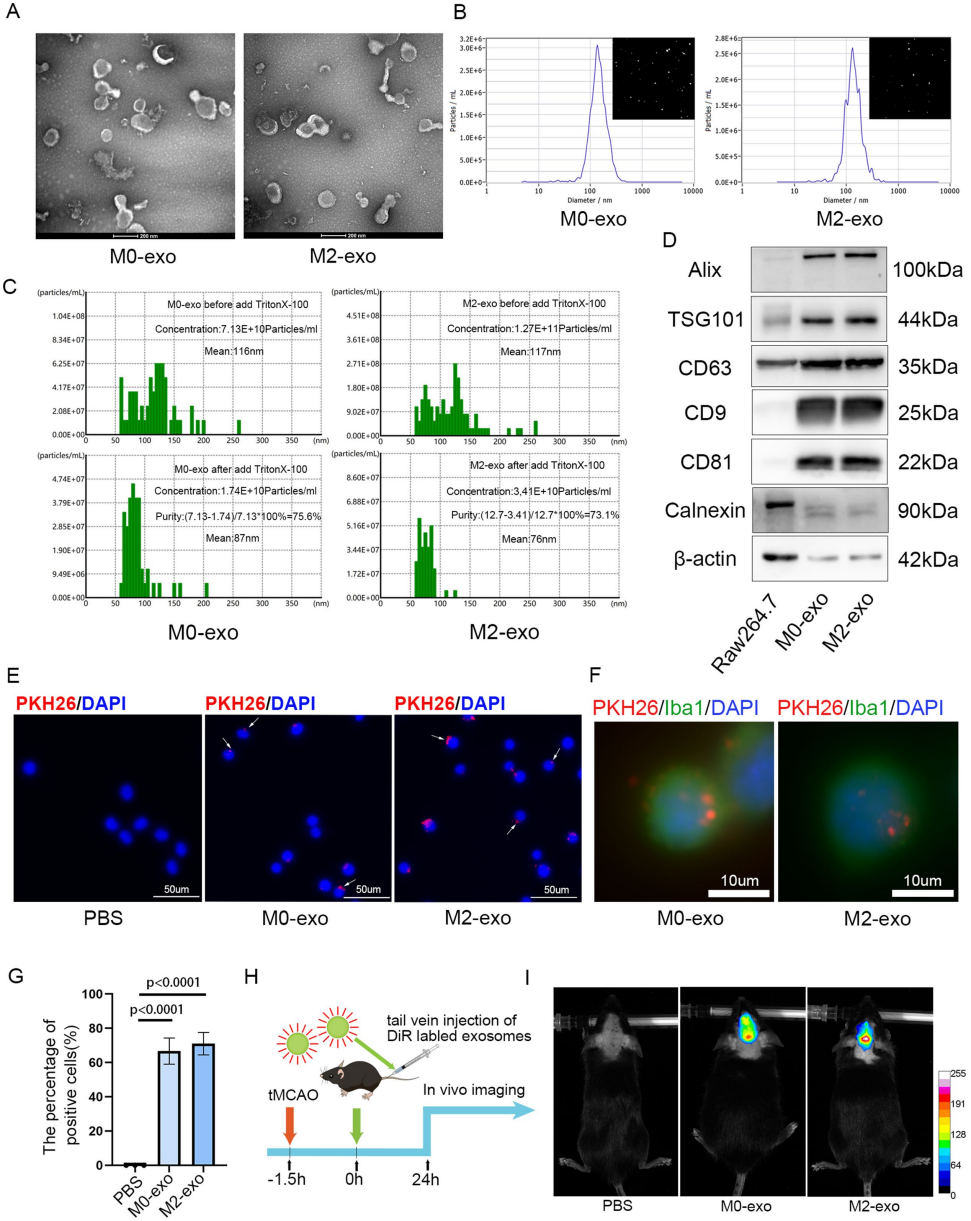
Characterization and uptake of M0-exo and M2-exo. **(A)** TEM images showing the biconcave disc morphology of M0-exo and M2-exo. Scale bar = 200 nm. **(B)** Concentration and size distribution of exosomes measured by NTA. **(C)** Exosome purity determined using NanoCoulter analysis. **(D)** WB analysis of exosome-specific markers. **(E–G)** Images showing the internalization of PKH26-labeled exosomes by BV2 cells (scale bars = 50 μm and 10 μm) and a bar graph quantifying the percentage of exosome-positive cells. Data are presented as the mean ± SD (*n* = 3). **(H)** A schematic diagram of the experimental workflow. **(I)** Fluorescence distribution observed in the brain 24 h after intravenous injection of DiR-labeled exosomes into tMCAO mice, visualized by an *in vivo* imaging system.

Exosomes were isolated from the culture supernatants of both M0 and M2 macrophages using differential ultracentrifugation. TEM revealed that the isolated exosomes displayed the typical saucer-shaped morphology (Figure 2A). NTA showed that M0-exo had a concentration of 1.3 × 10^10^ particles/mL with a median particle size of 140.8 nm, while M2-exo exhibited a concentration of 5.3 × 10^10^ particles/mL with a median size of 133.7 nm (Figure 2B). NanoCoulter analysis indicated purities of 75.6% for M0-exo and 73.1% for M2-exo (Figure 2C). WB analysis under equal protein loading conditions demonstrated that the exosome samples were enriched for the characteristic markers Alix, TSG101, CD63, CD9, and CD81, while Calnexin was nearly undetectable (Figure 2D). Both M0-exo and M2-exo were efficiently taken up by BV2 microglial cells *in vitro* (Figures 2E–G). *In vivo*, DiR-labeled exosomes were administered intravenously via the tail vein after filament removal in the tMCAO model. Fluorescent signals were observed in the ischemic brain 24 h post-injection, indicating successful exosome distribution (Figures 2H,I).

### 3.3 M2-exo exhibited a more potent anti-inflammatory effect compared to M0-exo in OGD/R BV2 microglial

**Figure 3:**
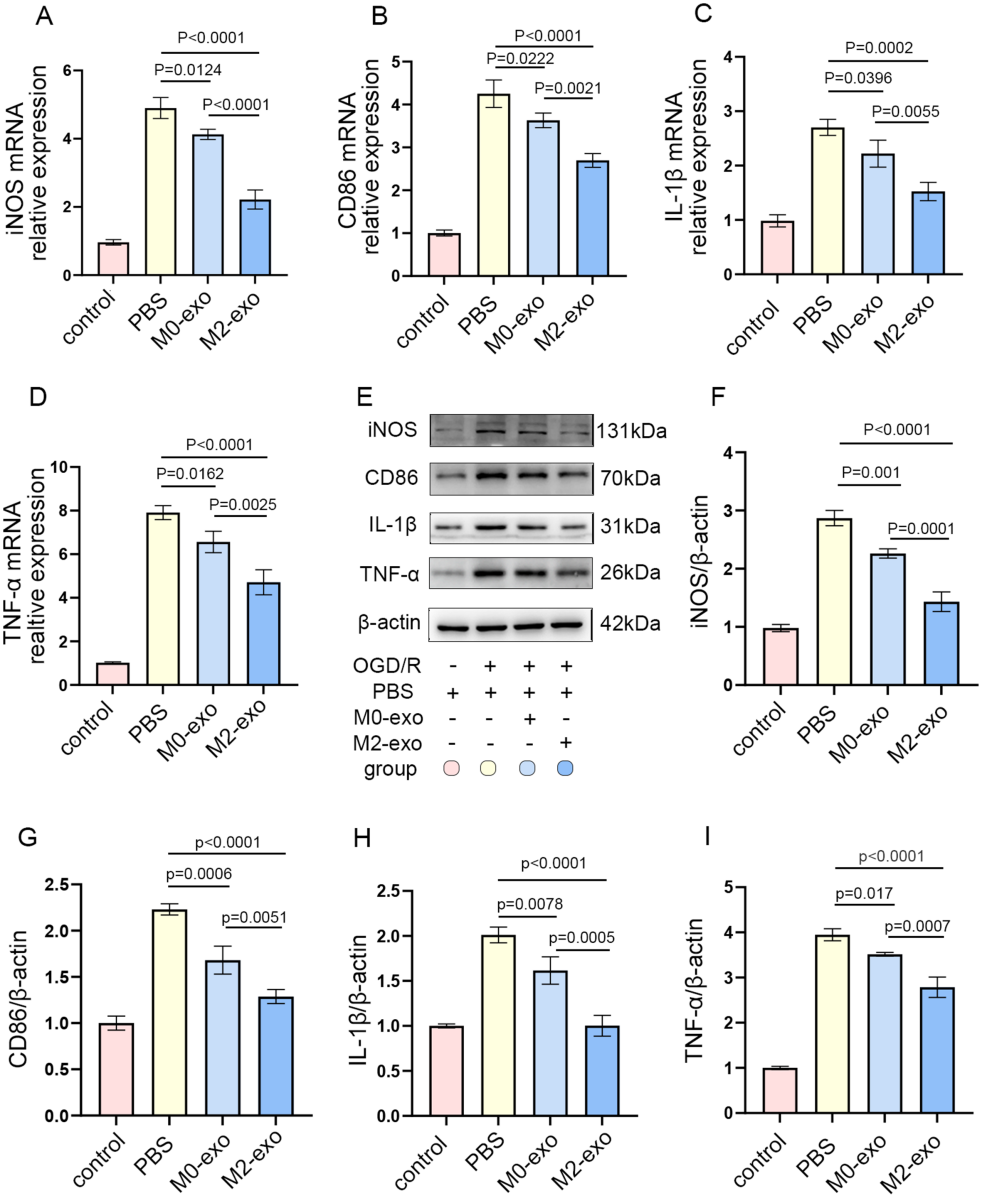
M2-exo demonstrated superior anti-inflammatory effects compared to M0-exo in BV2 cells subjected to OGD/R. **(A–D)** Relative mRNA expression of iNOS, CD86, IL-1-β, and TNF-*α* in each treatment group (*n* = 3). **(E–I)** Representative WB images and corresponding quantitative analyses of iNOS, CD86, IL-1-β, and TNF-α protein levels (*n* = 3). Data are presented as mean ± SD.

A concentration of 50 μg/mL is commonly used for exosome treatment *in vitro* (35, 36). BV2 microglial cells were treated with 50 μg/mL of exosomes at three time points: 2 h prior to OGD, during the 3 h OGD period, and throughout the 12 h reoxygenation phase. Cells were then harvested for qRT-PCR analysis. Both M0-exo and M2-exo treatment groups showed reduced mRNA expression levels of pro-inflammatory markers iNOS, CD86, IL-1β, and TNF-*α* compared to the PBS control group. Notably, the M2-exo group exhibited significantly lower expression of these cytokines than the M0-exo group (Figures 3A–D). To further validate the protein expression of pro-inflammatory factors, WB analysis was performed. The protein-level trends were consistent with the mRNA expression patterns observed via qRT-PCR (Figures 3E–I). These results demonstrate that both M0-exo and M2-exo confer anti-inflammatory effects in microglia following OGD/R, with M2-exo displaying superior efficacy.

### 3.4 M2-exo treatment significantly reduced infarct volume and attenuated the post-ischemic inflammatory response in tMCAO mice

**Figure 4:**
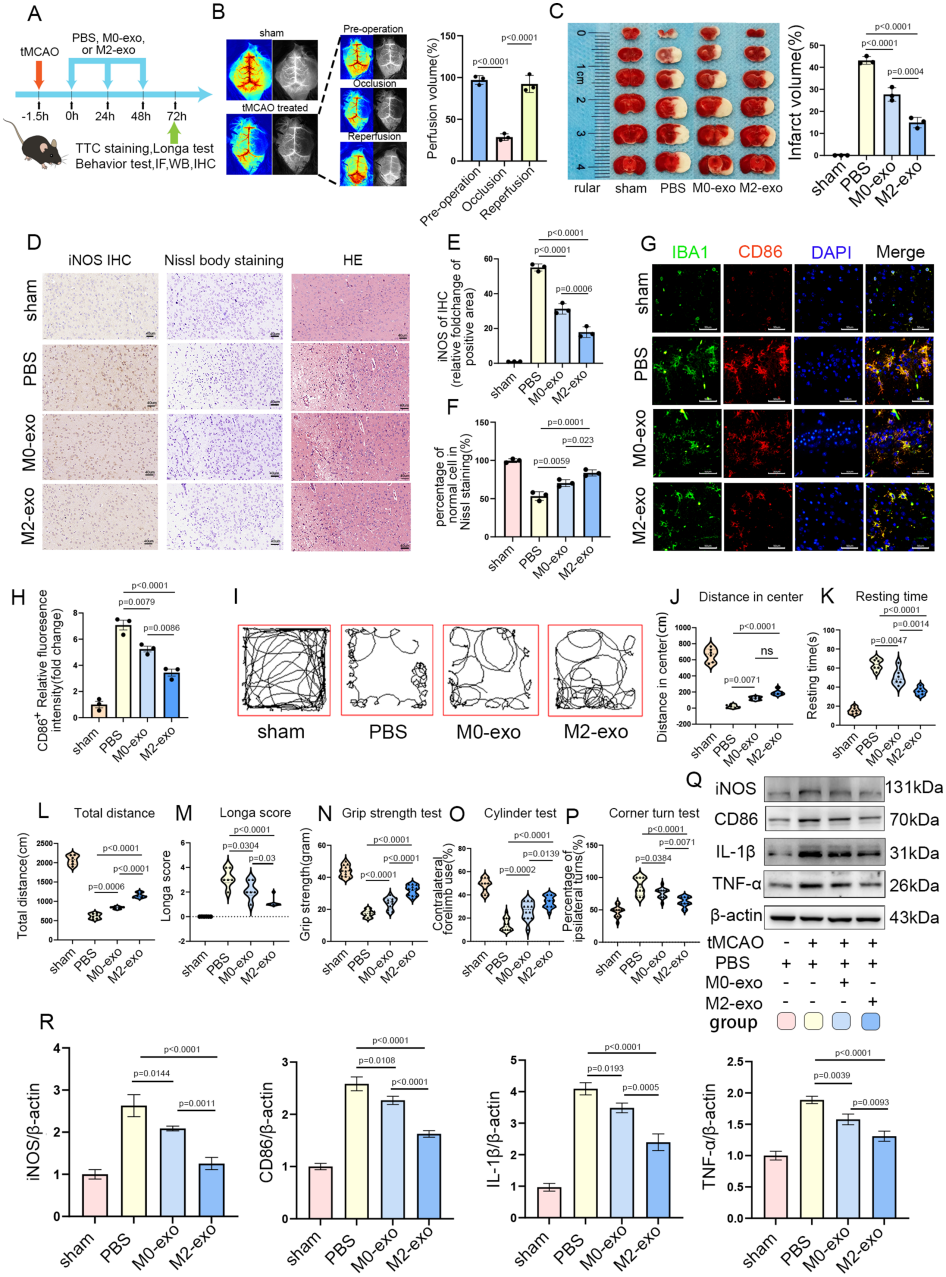
M2-exo exhibited superior neuroprotective effects compared to M0-exo. **(A)** Schematic overview of the experimental design. **(B)** LSI-based measurement of cerebral blood flow changes in sham and tMCAO mice (*n* = 3). **(C)** Representative TTC-stained brain sections and statistical analysis of infarct volume across groups (*n* = 3). **(D)** Representative images of iNOS immunohistochemistry, Nissl staining, and HE staining. Scale bar = 40 μm. **(E)** Quantitative analysis of iNOS immunohistochemistry (*n* = 3). **(F)** Statistical analysis of the percentage of normal neurons from Nissl staining (*n* = 3). **(G,H)** Representative CD86 immunofluorescence images and corresponding quantitative analysis (*n* = 3). Scale bar = 50 μm. **(I)** Representative movement paths recorded during the open field test. **(J–L)** Quantitative analysis of open field parameters: center distance (*n* = 6, cm), resting time (*n* = 6, s), and total distance traveled (*n* = 6, cm). **(M)** Longa neurological scores for each experimental group (*n* = 6). **(N–P)** Neurological function assessment via grip strength, cylinder, and corner turn tests (*n* = 6, with two measurements per mouse). **(Q,R)** WB images and quantitative analysis of inflammatory marker expression across groups (*n* = 3). Data are presented as mean ± SD.

Exosomes are commonly administered to mice at doses ranging from 100 to 200 μg per mouse (37, 38). The experimental timeline is depicted in Figure 4A. Both the M0-exo and M2-exo groups received daily intravenous injections of exosomes (7 μg/g body weight) for three consecutive days following tMCAO induction. The sham-operated and PBS control groups were administered an equivalent volume of PBS using the same regimen. LSI was utilized to monitor cerebral blood flow perfusion (39). A reduction in blood flow of ≥70% during filament occlusion, relative to baseline, was considered indicative of successful model induction (Figure 4B). TTC staining revealed that both M0-exo and M2-exo reduced cerebral infarct volume in tMCAO mice, with M2-exo exhibiting a significantly greater protective effect (Figure 4C).

Immunohistochemical analysis showed increased iNOS expression in the brain following cerebral infarction. Compared with M0-exo, M2-exo treatment more substantially reduced post-ischemic expression of the pro-inflammatory factor iNOS. Nissl staining revealed disintegrated and pyknotic neurons in the ischemic core, along with a reduction in Nissl bodies in surviving neurons within the ischemic penumbra. Nissl and hematoxylin–eosin (HE) staining demonstrated that administration of M0-exo or M2-exo preserved Nissl body integrity and maintained normal neuronal morphology, with M2-exo exerting more robust cytoprotective effects (Figures 4D–F).

Immunofluorescence double-labeling of Iba1 and CD86 showed that microglia in the ischemic penumbra exhibited altered morphology and elevated expression of the pro-inflammatory marker CD86. Exosome treatment mitigated this upregulation, and quantitative analysis revealed a more pronounced reduction in the M2-exo group (Figures 4G,H).

In the open field test, mice treated with exosomes demonstrated increased total movement distance and central zone exploration, along with significantly reduced immobility time, compared to the PBS group. The Longa scores were also lower in exosome-treated mice. Additionally, exosome treatment improved behavioral performance in the grip strength, cylinder, and corner turn tests. Notably, the M2-exo group exhibited superior behavioral outcomes compared to the M0-exo group (Figures 4I–P).

Finally, consistent with the findings shown in Figure 3, WB was used to assess the expression of inflammatory factors in brain tissues across the four experimental groups. The results indicated that intravenous administration of exosomes led to a marked downregulation of pro-inflammatory cytokines, including iNOS, CD86, IL-1β, and TNF-*α*, in the post-ischemic brain, confirming a substantial anti-inflammatory effect. Moreover, M2-exo demonstrated a stronger anti-inflammatory response than M0-exo (Figures 4Q,R).

### 3.5 M2-exo reduces the expression of Syk and Stat3 *in vitro* and *in vivo* by delivering miR-330-5p

**Figure 5:**
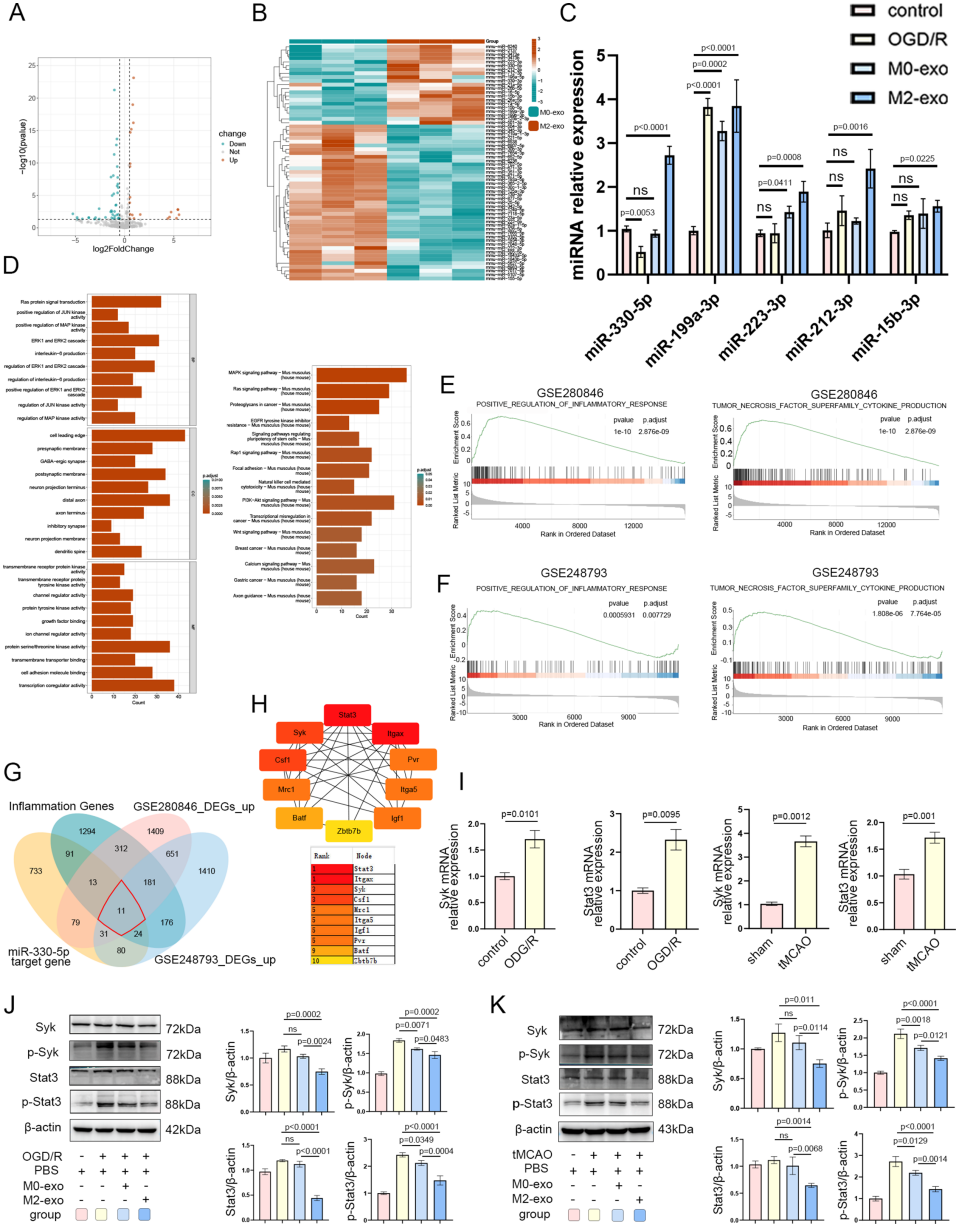
The superior anti-inflammatory activity of M2-exo is likely mediated by its packaged miR-330-5p. **(A)** Volcano plot of differentially expressed miRNAs in M0-exo vs. M2-exo (|log_2_FoldChange| ≥ 0.5, *p*-value < 0.05). **(B)** Heatmap showing expression patterns of differentially expressed miRNAs. **(C)** qRT-PCR validation of five candidate miRNAs across experimental groups (*n* = 3). **(D)** GO and KEGG enrichment analysis of predicted miR-330-5p target genes. **(E,F)** GSEA enrichment plots for datasets GSE280846 and GSE248793. **(G)** Venn diagram illustrating the intersection among four gene sets: 11 hub genes were identified. **(H)** Hub gene ranking based on node importance calculated using the MCC algorithm. **(I)** mRNA expression levels of Syk and Stat3 in BV2 microglial cells exposed to OGD/R (*n* = 3) and in the tMCAO mouse model (*n* = 3). **(J,K)** Representative WB images and quantitative analysis of Syk, p-Syk, Stat3, and p-Stat3 (*n* = 3). Data are presented as mean ± SD.

Given its superior anti-inflammatory efficacy, we focused on M2-exo to further investigate its underlying mechanism of action. High-throughput sequencing was performed to analyze miRNA cargo in both M0-exo and M2-exo. Differential expression analysis was conducted, and results were visualized using volcano plots and heatmaps, with thresholds set at |log_2_FoldChange| ≥ 0.5 and *p*-value < 0.05. A total of 62 differentially expressed miRNAs were identified, including 21 upregulated and 41 downregulated miRNAs (Figures 5A,B). Among the 21 upregulated candidates, 5 miRNAs with high log_2_FoldChange values were selected for further validation by qRT-PCR. The expression of miR-330-5p was found to be significantly downregulated under OGD/R conditions but markedly upregulated following M2-exo treatment. As it showed the most pronounced change, miR-330-5p was selected for subsequent analysis (Figure 5C).

To identify potential targets of miR-330-5p, we employed multiple prediction tools (DIANA-microT, miRDB, PicTar, PITA, and TargetScan), yielding 1,062 predicted target genes after duplicate removal. Subsequently, GO and KEGG pathway enrichment analyses were performed on these 1,062 target genes, revealing significant enrichment in protein tyrosine kinase activity, the MAPK signaling pathway, and interleukin-6 production (Figure 5D). Additionally, we performed differential expression analysis on the sham and MCAO groups from datasets GSE280846 and GSE248793 and visualized the results with volcano plots (Supplementary Figures 2A,B). GSEA of the two datasets revealed significant positive enrichment in the pathways “POSITIVE REGULATION OF INFLAMMATORY RESPONSE” and “TNF SUPERFAMILY CYTOKINE PRODUCTION” (Figures 5E,F).

The intersection of upregulated DEGs from GSE280846 and GSE248793 yielded 874 genes. GO and KEGG enrichment analyses of these 874 genes showed significant enrichment in pathways and biological processes such as myeloid leukocyte activation, macrophage migration, interleukin-6 production, and the TNF signaling pathway (Supplementary Figure 2C). These bioinformatic findings indicated that the predicted targets of miR-330-5p, as well as the infarction-induced upregulated genes, were significantly associated with inflammatory pathways.

To further refine potential regulatory targets, we obtained an inflammation phenotype-associated gene set from the GSEA database. After removing duplicates, we intersected these 2,102 inflammation-related genes with the upregulated DEGs from GSE280846, the upregulated DEGs from GSE248793, and the predicted target genes of miR-330-5p. This intersection identified 11 hub genes (Figure 5G). A PPI network of these 11 genes was constructed using the STRING database (Supplementary Figure 2D). Using the CytoHubba plugin in Cytoscape, we utilized the Maximal Clique Centrality algorithm to rank gene interactions. As shown in Figure 5H, Stat3 and Syk scored highly and are well-known mediators of inflammatory responses. In addition, a ceRNA regulatory network was visualized using Cytoscape to depict miRNA–hub gene interactions (Supplementary Figure 2E).

qRT-PCR analysis revealed that mRNA expression levels of both Syk and Stat3 were significantly upregulated under OGD/R conditions *in vitro* and following tMCAO *in vivo* (Figure 5I). WB analysis demonstrated that M2-exo treatment after OGD/R reduced the expression of Syk and Stat3, along with corresponding decreases in p-Syk and p-STAT3 levels in BV2 microglial cells (Figure 5J). Similarly, intravenous M2-exo administration following cerebral ischemia in mice resulted in downregulation of both total and phosphorylated Syk and Stat3 in brain tissue (Figure 5K). Collectively, these results preliminarily demonstrate that the inflammation-alleviating effect of M2-exo may be mediated by its cargo miR-330-5p through the targeting of Syk and Stat3.

### 3.6 miR-330-5p exerts its anti-inflammatory effects by targeting Syk and Stat3

**Figure 6:**
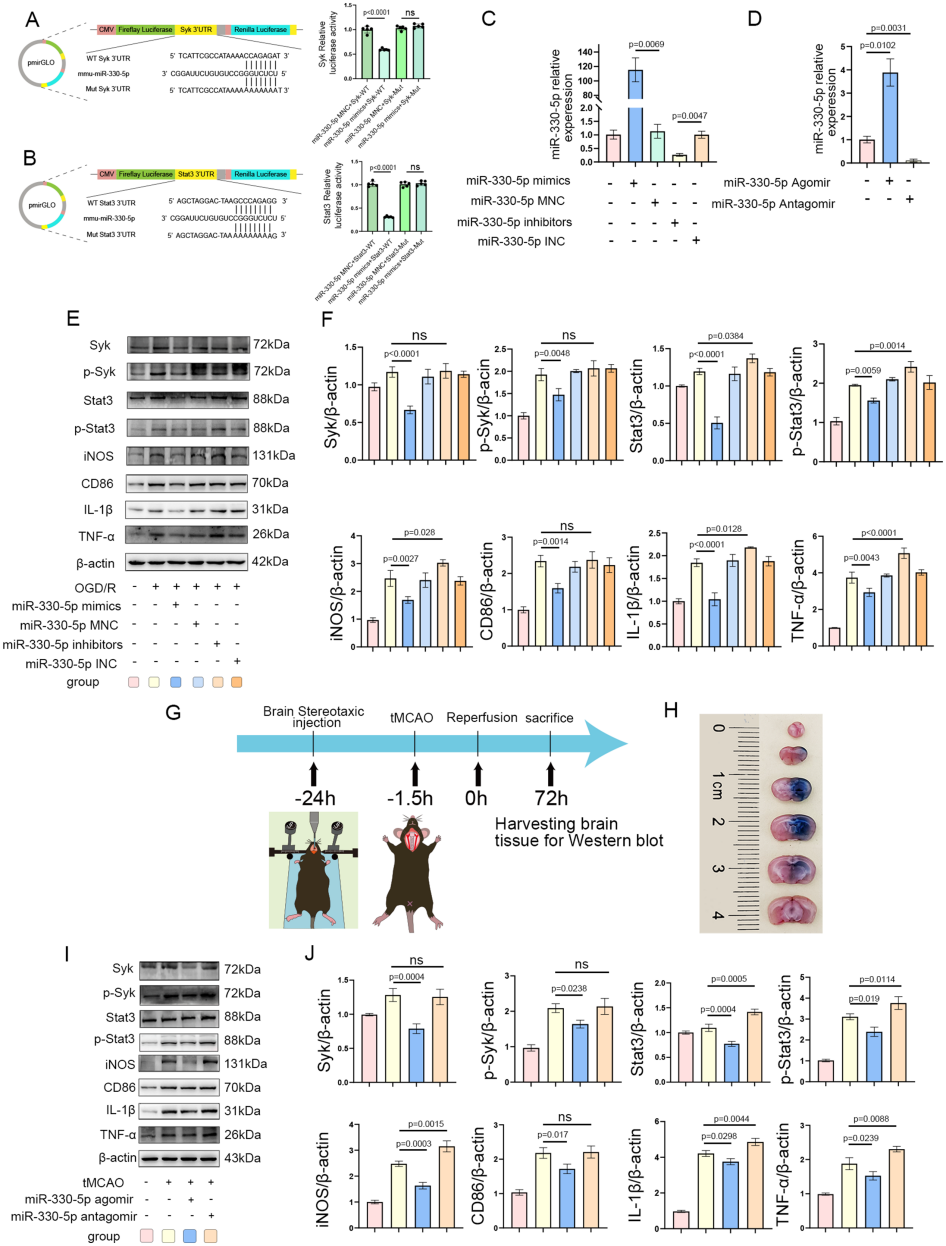
Syk and Stat3 are direct targets of miR-330-5p. **(A,B)** The miR-330-5p mimics + Syk(or Stat3)-Wild Type (WT) group exhibited a significant decrease in fluorescence intensity compared to the miR-330-5p mimics negative control (MNC) + Syk(or Stat3)-WT group. The miR-330-5p mimics + Syk(or Stat3)-Mutant type (Mut) group showed no significant change in fluorescence intensity compared to the miR-330-5p MNC + Syk(or Stat3)-Mut group. In summary, miR-330-5p suppresses firefly luciferase activity by binding to the 3′-untranslated regions (UTRs) of Syk and Stat3 (*n* = 5). **(C)** Transfection of BV2 microglial cells with miR-330-5p mimics significantly increased miR-330-5p expression compared to the mimics negative control (MNC) group. Conversely, transfection with miR-330-5p inhibitors significantly decreased its expression relative to the inhibitors negative control (INC) group (*n* = 3). **(D)** MiR-330-5p expression in brain tissues of mice from each group (*n* = 3). **(E,F)** Representative WB images and quantitative analysis of target proteins (*n* = 3). **(G)** Schematic of the stereotaxic intracerebral injection of agomir and antagomir in mice. **(H)** Distribution of Evans Blue in the brain 24 h after stereotaxic injection into the striatum. **(I,J)** Representative WB images and quantification of target protein expression (*n* = 3). Data are presented as mean ± SD.

Dual-luciferase reporter assays confirmed the interaction between miR-330-5p and the 3’-Untranslated Regions (3’-UTRs) of Syk and Stat3, resulting in decreased firefly luciferase activity (Figures 6A,B). The transfection efficiency of miR-330-5p mimics, inhibitors, and their respective negative controls (MNC and INC) was validated by qRT-PCR. A marked upregulation of miR-330-5p was observed in the mimics group relative to both the untransfected and negative control groups, while the inhibitor group showed a reduction in expression to approximately 30% of baseline levels (Figure 6C). In mouse experiments, stereotaxic intracerebral injection of the miR-330-5p agomir resulted in an approximately fourfold increase in miR-330-5p expression compared to the control group, whereas the antagomir group exhibited a significant reduction in expression (Figure 6D).

WB analysis demonstrated that in OGD/R-treated BV2 microglial cells, transfection with miR-330-5p mimics downregulated the expression of Syk, Stat3, and their phosphorylated forms (p-Syk and p-Stat3), along with reductions in the inflammatory mediators TNF-*α*, IL-1β, CD86, and iNOS. In contrast, transfection with the inhibitor upregulated the expression of Stat3, p-Stat3, TNF-α, IL-1β, and iNOS (Figures 6E,F). Figure 6G illustrates the experimental design for miR-330-5p overexpression or inhibition *in vivo*. Evans Blue staining showed that 24 h after stereotaxic injection, the tracer had diffused throughout the right striatum and cortex, overlapping with the infarcted region and confirming effective agent delivery (Figure 6H). Intracerebral injection of miR-330-5p agomir reduced the expression of Syk, Stat3, p-Syk, p-Stat3, and the inflammatory mediators TNF-α, IL-1β, CD86, and iNOS, while antagomir administration induced the opposite pro-inflammatory effect (Figures 6I,J).

### 3.7 Administration of Syk and Stat3 inhibitors mimicked the anti-inflammatory effects of miR-330-5p

**Figure 7:**
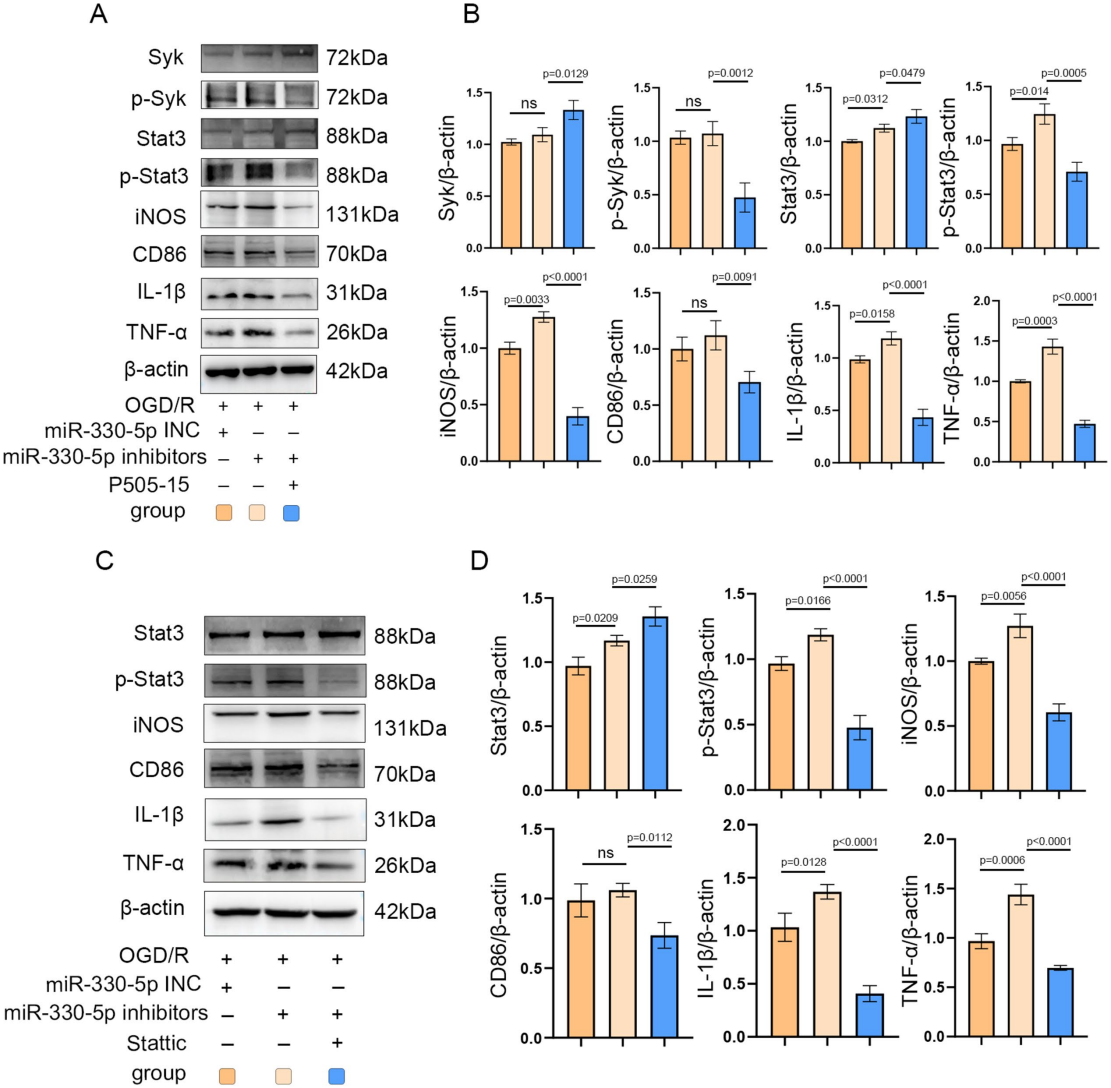
Administration of the Syk inhibitor P505-15 and the Stat3 inhibitor Stattic mimicked the anti-inflammatory effects of miR-330-5p. **(A–D)** Representative WB images and quantitative analysis of target proteins (*n* = 3). Data are presented as mean ± SD.

To further confirm the role of Syk and Stat3 in miR-330-5p-mediated anti-inflammatory effects, a rescue experiment was conducted. After being transfected with the miR-330-5p inhibitor, BV2 microglia were treated with the Syk inhibitor P505-15. The results demonstrated that Syk inhibition effectively mimicked the anti-inflammatory action of miR-330-5p, as evidenced by reduced phosphorylation of Syk and its downstream target Stat3. Accordingly, the production of iNOS, CD86, TNF-α, and IL-1β were notably decreased (Figures 7A,B). Furthermore, BV2 cells transfected with the miR-330-5p inhibitor were treated with the Stat3 inhibitor Stattic. Stattic treatment led to a marked decrease in Stat3 phosphorylation and was accompanied by reduced expression of downstream inflammatory markers (Figures 7C,D). These results collectively establish that miR-330-5p targets both Syk and Stat3 to exert anti-inflammatory effects via the Syk/Stat3 signaling pathway.

## 4 Discussion

An excessive inflammatory response during the acute phase of cerebral infarction is a critical factor contributing to aggravated brain injury. The acute inflammatory reaction typically peaks 3–5 days after stroke onset, during which activated microglia release large quantities of inflammatory mediators, triggering an inflammatory cascade (40). This study aimed to identify strategies that mitigate this excessive inflammation during the acute phase and, in doing so, explore potential therapeutic targets for ischemic stroke. Macrophage membrane-derived vesicles demonstrate efficient accumulation in the infarcted cerebral region, thereby enabling the delivery of molecular cargo across the blood–brain barrier and facilitating partially targeted drug delivery (41). Prior studies have reported that M2-exo exerts protective effects in models of OS (38, 42, 43). Based on this evidence, we selected macrophage-derived exosomes as a promising therapeutic target for further investigation.

Macrophages can be polarized to the M2 phenotype by key stimuli, including the cytokines IL-4 and IL-13 (44). In our study, qRT-PCR, WB, IF, FCM, and morphological assessments confirmed that co-treatment with IL-4 and IL-13 successfully polarized Raw 264.7 macrophages into the M2 subtype. This polarization was a necessary prerequisite for the subsequent isolation of M2-exosomes. The exosomes were characterized by TEM, NTA, NanoCoulter analysis, and WB to evaluate morphology, particle size, concentration, purity, and expression of specific marker proteins. Rigorous quality control is essential, as consistent exosome quality underpins their biological activity. PKH26 and DiR labeling were used to trace exosome uptake *in vitro* and *in vivo*, respectively (31, 45). Our results demonstrate that exosomes effectively reach the target site both *in vivo* and *in vitro*.

iNOS, CD86, IL-1β, and TNF-*α* are commonly used as key markers for evaluating the inflammatory response after ischemic stroke (46, 47). Following ischemic insult, microglial activation leads to marked upregulation of iNOS, CD86, IL-1β, and TNF-α in M1-polarized microglia. This elevated inflammatory response contributes to further brain damage, whereas suppression of these pro-inflammatory mediators has been shown to exert neuroprotective effects (48, 49). In our experiments, both M0-exo and M2-exo significantly reduced the expression of iNOS, CD86, IL-1β, and TNF-α following OS induction *in vitro* and *in vivo*. Notably, M2-exo demonstrated superior anti-inflammatory efficacy compared to M0-exo. In line with this reduction in pro-inflammatory cytokines, M2-exo also conferred more substantial neuroprotection in the tMCAO mouse model.

To elucidate the mechanism underlying the enhanced anti-inflammatory effects of M2-exo relative to M0-exo, we performed high-throughput miRNA sequencing on both exosome types. Differential expression analysis identified 21 miRNAs significantly upregulated in M2-exo. Among these, the five most promising candidates were selected for validation via qRT-PCR. We found that miR-330-5p was downregulated in BV2 microglial cells following OGD/R. However, this downregulation was reversed by M2-exo treatment, suggesting that M2-exo delivers miR-330-5p into microglia under stress conditions. It has been reported that miR-330-5p alleviates inflammation and reduces myocardial injury by targeting TIM3 during ischemia–reperfusion injury (50). Furthermore, evidence indicates that mesenchymal stem cell-derived exosomes attenuate inflammation in a rat model of ischemic stroke by delivering miR-330-5p, which targets SETD7 (51). miR-330-5p has also been reported to promote M2 polarization of microglia while inhibiting the M1 phenotype (52). Whether exosomes derived from M2 macrophages, which are rich in miR-330-5p, have a therapeutic effect on ischemic stroke has not been reported. Based on our sequencing and qPCR results, combined with previous literature, we choose miR-330-5p as a key molecule for further investigation. The predicted target genes of miR-330-5p were significantly enriched in multiple inflammation-related pathways. Similarly, upregulated DEGs from public datasets GSE280846 and GSE248793 were also strongly enriched in inflammatory signaling pathways. Using Venn diagram analysis, we identified 11 hub genes. Stat3 and Syk were top-ranked by MCC scoring and were selected as key genes for mechanistic analysis.

Syk plays a critical role in inflammatory processes. In macrophages, it promotes inflammatory responses by activating NF-κb (53). Activation of Syk drives microglial polarization toward a pro-inflammatory phenotype (54). Inhibition of Syk activation during mesenteric ischemia–reperfusion has been shown to reduce both local intestinal inflammation and remote lung injury (55). It has not been reported whether miR-330-5p exerts an anti-inflammatory effect by targeting Syk. Thus, it is of great interest to explore the functional role of miR-330-5p derived from M2-exosomes in the suppression of Syk.

Stat3 is similarly involved in inflammatory processes, orchestrating their initiation and progression. In microglia, Stat3 deficiency alleviates neuroinflammation and improves neurological outcomes following subarachnoid hemorrhage in mice (56). In renal ischemia–reperfusion injury, inhibition of Stat3 activation exerts a protective effect (57). Blocking the JAK/Stat3 pathway also reduces infarct size and suppresses inflammatory cytokine expression in rats (58, 59). Under OGD/R conditions, Stat3 activation promotes M1 polarization of microglia (60), whereas inhibition of Stat3 phosphorylation facilitates M2 polarization (61). Previous studies have identified Stat3 as a target gene of miR-330-5p. In a mouse model of spinal cord injury, miR-330-5p directly targets and downregulates Stat3, leading to decreased iNOS production (62). Additionally, HuR has been shown to promote Stat3 protein expression by interfering with miR-330-5p binding to the 3′-UTR of Stat3 mRNA (63). Our study shows that M2-exo treatment reduces the protein levels of Syk, Stat3, p-Syk, and p-Stat3 in both OGD/R-treated microglial cells and the tMCAO mouse model, as determined by Western blot analysis.

The targeting of Syk and Stat3 3′-UTRs by miR-330-5p was established using dual-luciferase reporter assays. In BV2 cells transfected with miR-330-5p mimics, the expression levels of Syk and Stat3 were lower than those in the negative control group, and corresponding levels of p-Syk, p-Stat3, CD86, TNF-*α*, IL-1β, and iNOS were also reduced. The mimics exerted a significant anti-inflammatory effect, whereas the inhibitor-transfected group showed the opposite pro-inflammatory response. Similarly, *in vivo*, mice injected with miR-330-5p agomir exhibited reduced expression of Syk and Stat3 compared to the PBS group, thereby attenuating the inflammatory response.

We propose that this anti-inflammatory effect arises from two synergistic mechanisms. First, miR-330-5p targets and inhibits Syk, thereby reducing its phosphorylation and subsequent inflammatory signaling—consistent with prior reports showing that inhibition of Syk activation alleviates inflammation (13, 14, 55, 64). Moreover, Syk activation influences Stat3 activation. During OS, Syk is essential for Stat3 phosphorylation (65), and enhanced Syk activity facilitates phosphorylation of its downstream target, Stat3 (23, 66). Thus, miR-330-5p may alleviate inflammation by targeting Syk, decreasing its expression, and thereby suppressing the Syk/Stat3 signaling pathway. Second, our experiments verified that miR-330-5p can directly target Stat3 to reduce its protein expression. This is consistent with earlier studies demonstrating that miR-330-5p binds the 3′-UTR of Stat3 mRNA (62, 63). Activated p-Stat3 sustains NF-κB activity by prolonging its nuclear retention time (67). Once activated, NF-κB enhances the transcription of various inflammatory mediators (68). PTN has been reported to reduce microglial activation and attenuate inflammation by inhibiting the Stat3/NF-κB pathway (69). Reducing nuclear p-Stat3 levels can suppress the expression of downstream pro-inflammatory factors (70). Based on our results, miR-330-5p reduces the secretion of these factors by targeting Stat3 and decreasing p-Stat3 levels. This dual mechanism—miR-330-5p targeting both Syk and Stat3—may underlie the superior anti-inflammatory efficacy of M2-exo compared to M0-exo.

The Syk inhibitor P505-15 (10 μM) has been reported to reduce microglial phagocytosis of neurons, thereby exerting a neuroprotective effect (71). To conduct a rescue validation, we administered P505-15 (10 μM) to microglial cells transfected with a miR-330-5p inhibitor and subjected to OGD/R injury. Administration of P505-15 partially reversed the pro-inflammatory effects induced by miR-330-5p inhibition. Additionally, we performed a rescue experiment targeting Stat3 to further confirm its role in this pathway. Stattic, a potent Stat3 inhibitor, functions by blocking Stat3 phosphorylation (69) and has been shown to alleviate LPS-induced inflammation in macrophages (72). In our study, Stattic administration similarly mitigated the pro-inflammatory effects elicited by miR-330-5p inhibition. Taken together, our integrated findings demonstrate that M2-exo exerts its anti-inflammatory effects through the delivery of miR-330-5p, which mediates targeted inhibition of both Syk and Stat3.

Our work is the first to demonstrate through high-throughput sequencing that miR-330-5p is highly enriched in exosomes derived from M2 macrophages. Second, we provide the first evidence that miR-330-5p can directly target Syk and alleviate inflammatory responses by downregulating Syk expression. Third, we revealed a novel mechanism whereby miR-330-5p, enriched in M2-derived exosomes, exerts a significant therapeutic effect on the acute inflammatory response following ischemic stroke via the Syk/Stat3 signaling pathway.

This study has several limitations. First, although both M0-exo and M2-exo displayed anti-inflammatory properties, our mechanistic investigations focused exclusively on M2-exo due to their superior efficacy. The molecular mechanisms underlying the anti-inflammatory effects of M0-exo remain to be elucidated. Second, our rescue experiments demonstrated that Syk activation exerts an anterograde effect on Stat3 activation. However, whether Stat3 reciprocally modulates Syk expression remains to be investigated. Third, it is established by prior work that activation of the Stat3 signaling pathway promotes angiogenesis and contributes to long-term functional recovery following ischemic stroke (73–75). Activation of Stat3 promotes neurogenesis during the recovery phase of cerebral infarction (76, 77). There seems to be a contradiction between the outcomes of these studies and our results. We hypothesize that this discrepancy may stem from the temporal dynamics of Stat3 activity during stroke pathology. Specifically, overactivation of Stat3 in the acute phase exacerbates inflammation, while in the chronic phase, Stat3 activation promotes tissue repair via angiogenesis and neurogenesis. Long-term suppression of Stat3 may adversely affect repair processes during the chronic phase of ischemic stroke. To avoid this risk, we propose that the administration of M2 macrophage-derived exosomes should be limited to the acute phase (within 72 h) post-infarction. This timed intervention aims to suppress excessive inflammation and alleviate oxidative stress during the acute phase, without compromising long-term neurological recovery. Our short-term findings, which demonstrate the alleviation of inflammation via SYK and STAT3 inhibition within 72 h, constitute a critical first step within this broader physiological context. The long-term effects of M2-exosome administration after cerebral infarction warrant further investigation. Finally, we isolated exosomes from the Raw264.7 cell line, which cannot fully recapitulate all physiological functions of primary macrophages. Our study represents a preliminary exploration of M2-exosome application. Whether M2-exosomes derived from primary macrophage induction possess similar or superior functions warrants further investigation.

## 5 Conclusion

**Figure 8:**
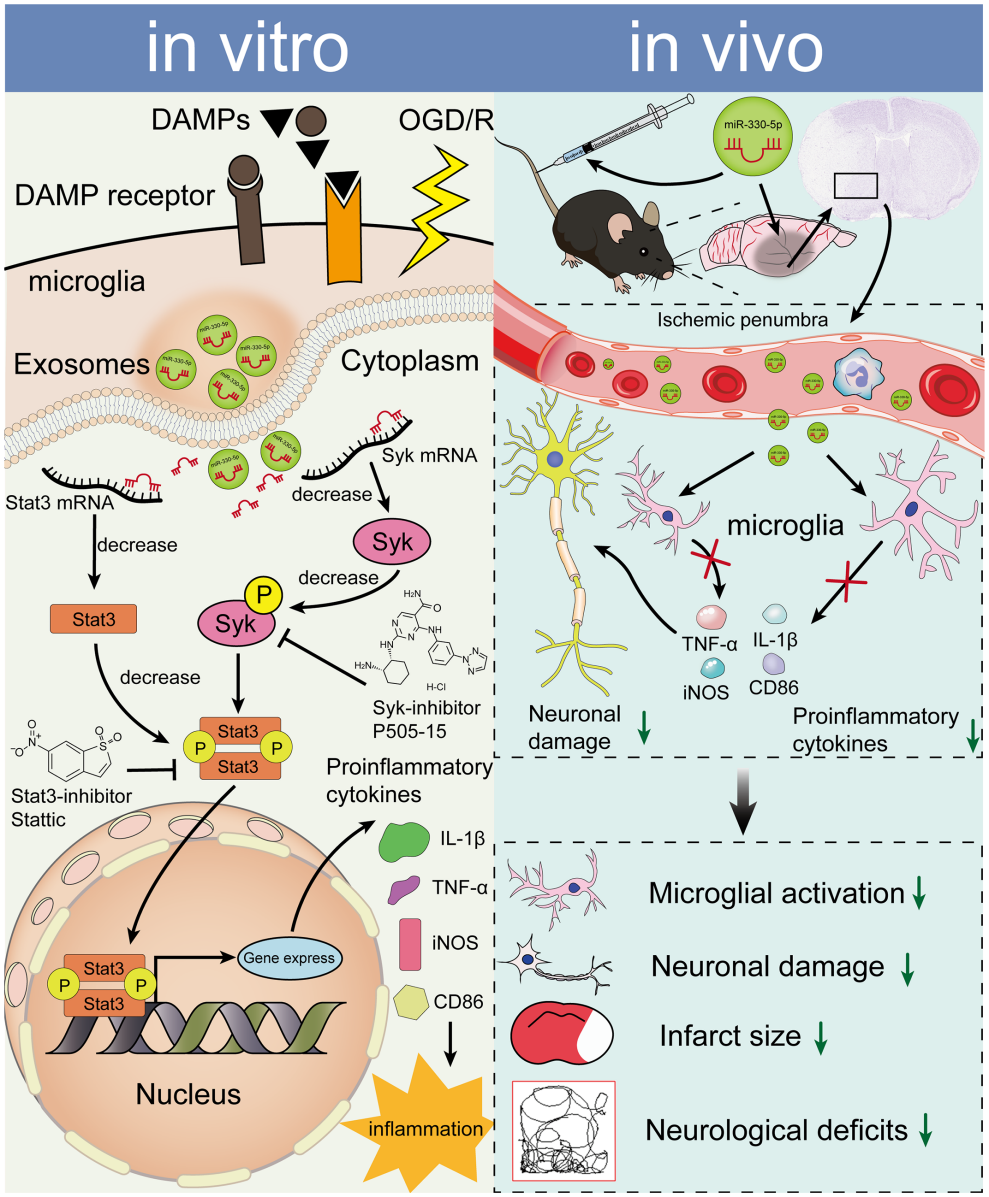
miR-330-5p, enriched in M2-exo, mediates an anti-inflammatory response via suppression of the Syk/Stat3 signaling pathway, ultimately reducing inflammatory factor expression in both OGD/R-treated BV2 microglia and the tMCAO mouse model.

In summary, M2-exo exhibited superior anti-inflammatory effects compared to M0-exo in the inflammatory response following OS. This effect is mechanistically mediated through the miR-330-5p/Syk/Stat3 signaling axis (Figure 8).

## Data Availability

The original contributions presented in the study are publicly available. This data can be found here: https://www.ncbi.nlm.nih.gov/, accession number GSE312882.

## Footnotes

1
https://fiji.sc

2
http://www.r-project.org

3
http://cn.string-db.org

4
http://cytoscape.org
