# Supplementary figures and images for: M2 macrophage-derived exosomes mitigate acute inflammation following ischemic stroke
Source: Front Neurol. 2026 Feb 4;17:1733679. doi: 10.3389/fneur.2026.1733679 (PMC12913138; doi:10.3389/fneur.2026.1733679)

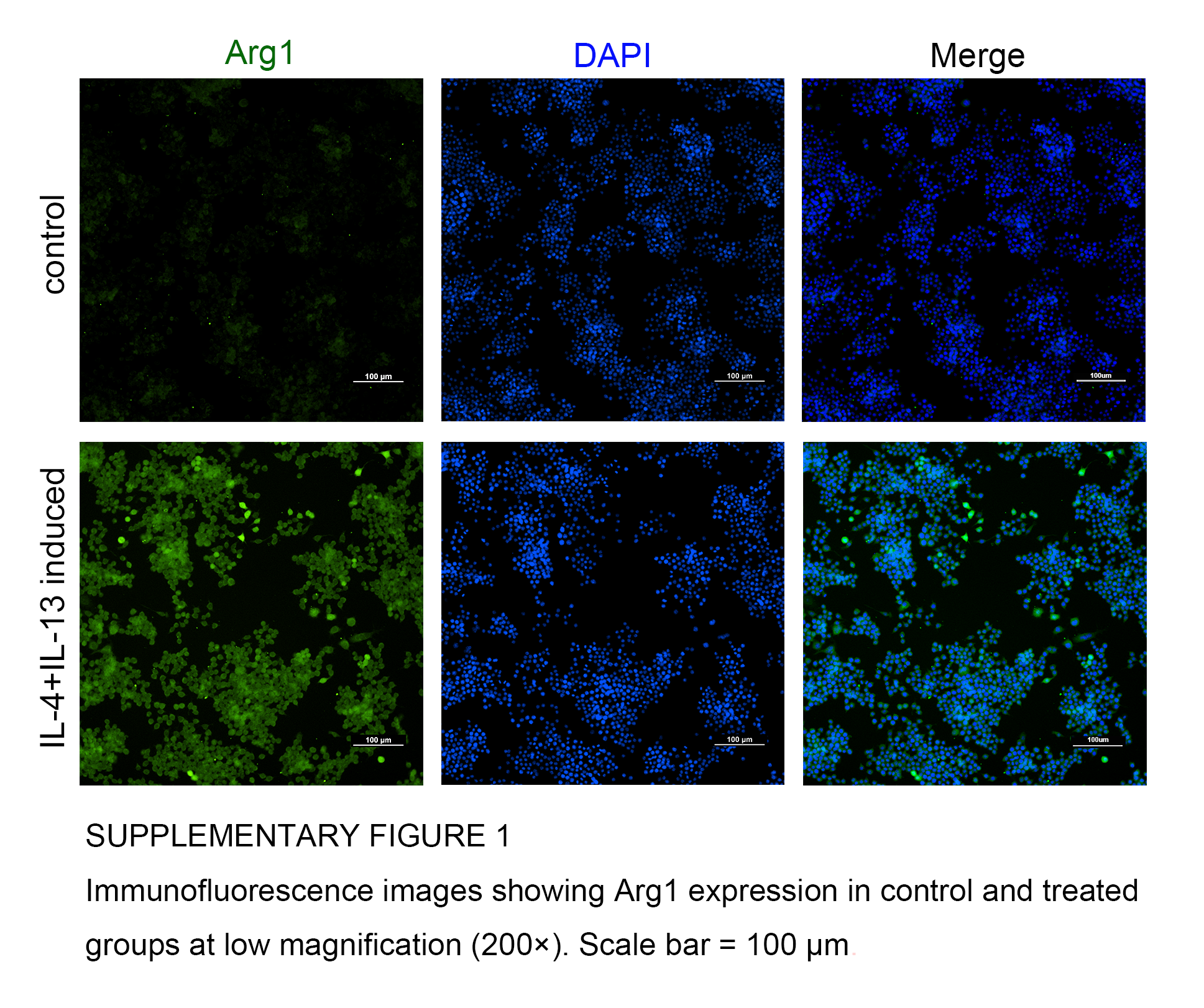

Supplement: Supplementary file 1 [file Image_1.tif]

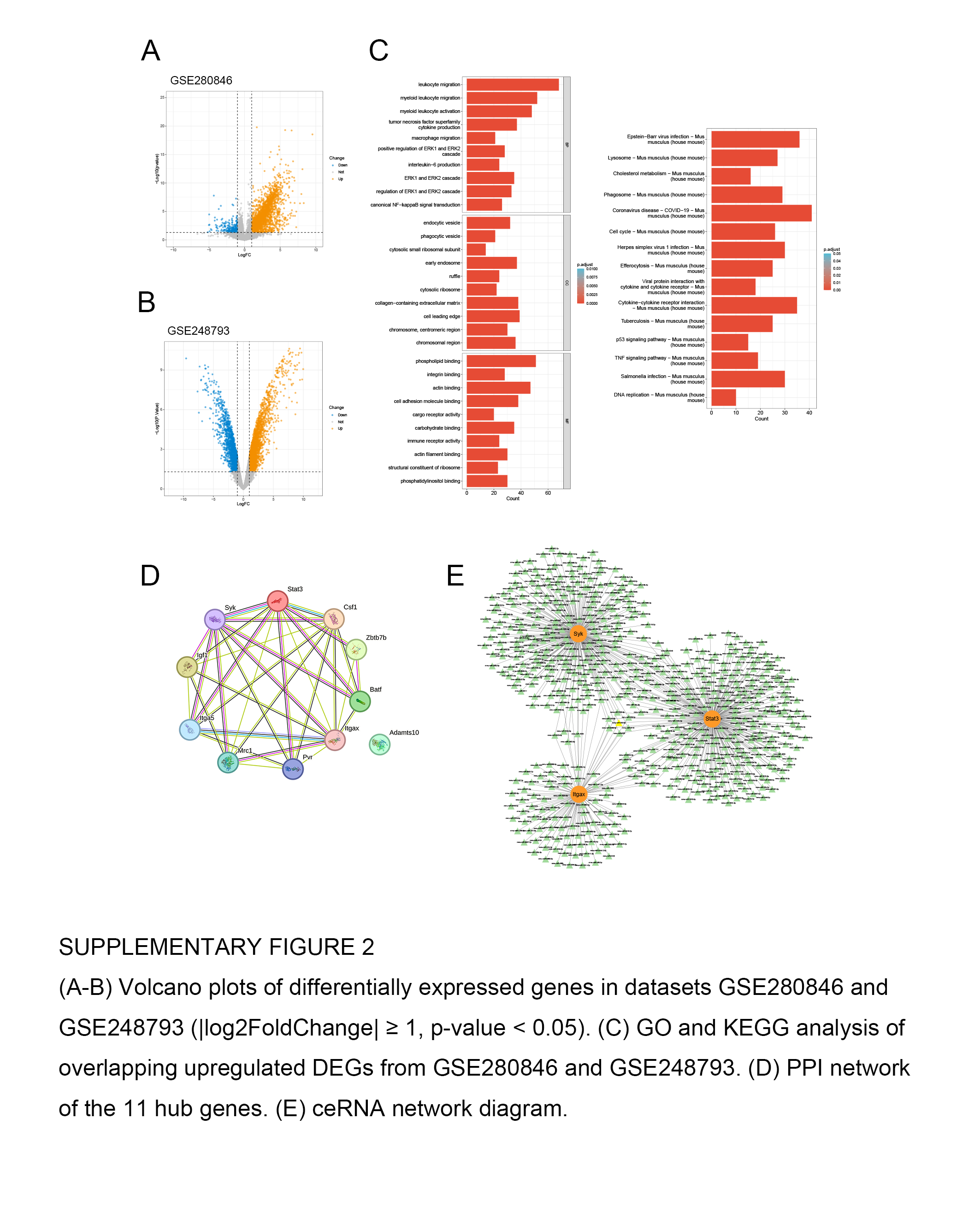

Supplement: Supplementary file 2 [file Image_2.tif]
